# Salicylate Toxicity from Genital Exposure to a Methylsalicylate-Containing Rubefacient

**DOI:** 10.5811/westjem.2016.1.29262

**Published:** 2016-03-02

**Authors:** Trevonne M. Thompson, Theodore Toerne, Timothy B. Erickson

**Affiliations:** * The University of Illinois at Chicago, Department of Emergency Medicine, Chicago, Illinois; † Advocate Christ Medical Center, Department of Emergency Medicine, Oak Lawn, Illinois

## Abstract

Methylsalicylate-containing rubefacients have been reported to cause salicylate poisoning after ingestion, topical application to abnormal skin, and inappropriate topical application to normal skin. Many over-the-counter products contain methylsalicylate. Topical salicylates rarely produce systemic toxicity when used appropriately; however, methylsaliclyate can be absorbed through intact skin. Scrotal skin can have up to 40-fold greater absorption compared to other dermal regions. We report a unique case of salicylate poisoning resulting from the use of a methylsalicylate-containing rubefacient to facilitate masturbation in a male teenager. Saliclyate toxicity has not previously been reported from the genital exposure to methylsaliclyate.

## INTRODUCTION

Many over-the-counter products are available to treat common musculoskeletal aches and pains. Often these products are in the form of ointments, liniments, and rubefacients. Methylsalicylate is an active ingredient in many of the over-the-counter rubefacients. Methylsalicylate-containing rubefacients have been reported to cause salicylate poisoning after ingestion, topical application to abnormal skin, and inappropriate topical application to normal skin.^1^–^5^ Many over-the-counter products contain methylsalicylate because of its mild analgesic and anti-inflammatory properties. These over-the-counter products may contain up to 30% methylsalicylate. In the United States, drug preparations containing >5% methylsalicylate must bear a label warning against misdirected use and that the product should be kept out of the reach of children.^6^ We present a unique case of salicylate poisoning caused by genital exposure to a methylsalicylate-containing rubefacient used to facilitate masturbation in a teenager.

## CASE REPORT

A 14-year-old male presented to the emergency department (ED) complaining of shortness of breath, chest pain, lightheadedness, vomiting, and malaise. He noticed the symptoms earlier in the day when he was preparing for school. The patient was afebrile with heart rate of 100 beats per minute, blood pressure of 100/60mmHg, respiratory rate of 30 per minute, and a pulse oximetry measurement of 100% on room air. The remainder of the physical examination was pertinent for tachypnea with clear lungs. The cardiovascular and gastrointestinal examinations were normal. Laboratory evaluation revealed a serum glucose of 290mg/dL, serum bicarbonate of 15mEq/L, and an anion gap of 21. The patient was presumed to have new-onset diabetes with diabetic ketoacidosis. He was given a normal saline bolus, started on an insulin infusion, and transferred to a pediatric hospital. Upon arrival to the referral hospital, the patient had normal glucose measurements, and the insulin infusion was stopped. An arterial blood gas measurement revealed a pH of 7.44, pCO_2_ of 18mm/Hg, and a bicarbonate of 12mEq/L. As a result of his acid-base status and tachypnea, there was concern for salicylate poisoning as the source of the abnormalities. A salicylate concentration was ordered and returned at 68mg/dL. Since there was no history of salicylate exposure, the measurement was repeated about four hours later and returned at 63mg/dL. A bicarbonate infusion was started to treat salicylate poisoning while further questioning of the patient ensued.

On direct questioning, the patient denied ingesting aspirin or salicylate-containing products but eventually admitted to using an entire 60-gram tube of BENGAY^®^, which contains methylsalicylate, to facilitate masturbation the day prior to the ED presentation. The patient was successfully treated with a sodium bicarbonate infusion and supportive care for the salicylate poisoning. There was no indication for hemodialysis. The patient was discharged four days after initial presentation in stable condition.

## DISCUSSION

Salicylic acid is a cellular poison that indiscriminately impairs cellular metabolism in overdose.^7^ The clinical presentation of salicylate poisoning is related to stimulation of the central nervous system respiratory center, disturbance of lipid and carbohydrate metabolism, and disturbance of intracellular respiration.^8^ Symptoms can include hyperpnea, tachypnea, tinnitus, hyperpyrexia, and diaphoresis.^8^ Additional signs are dehydration, electrolyte disturbances, serum glucose abnormalities, and mixed acid-base disturbances.^8^ More severe toxicity can result in acute lung injury, lethargy, coma, seizures, cerebral edema, and death.^8^ Treatment of salicylate poisoning consists of general supportive care, gastrointestinal decontamination with activated charcoal in cases of salicylate ingestion, and monitoring of serum salicylate concentrations. Enhanced elimination of salicylate is achieved by a sodium bicarbonate infusion with maintenance of serum potassium homeostasis or hemodialysis if the bicarbonate infusion is ineffective or cannot be performed.

Methylsalicylate, also known as oil of wintergreen, is widely available in many over-the-counter ointments, lotions, and salves for the relief of musculoskeletal aches and pains.^9^ The table provides examples of products that contain various concentrations of methylsalicylate. In vivo, methylsalicylate is hydrolized to salicylic acid. Five milliliters of oil of wintergreen is approximately equal to 7,000mg of salicylate or twenty-two 325mg aspirin pills.^9^ Topical salicylates rarely produce systemic toxicity when used appropriately; however, methylsaliclyate can be absorbed through intact skin.^10^ Exercise and heat exposure can enhance percutaneous absorption of methylsaliclyate.^11^ At least one death has been reported in an athlete using an excessive amount of a methylsalicylate-containing rubefacient.^5^ There can be increased absorption of methylysalicylate with abnormal skin such as psoriasis, erythroderma, and burns.^12^,^13^ There can also be increased aborption of methylsalicylate when applied to large areas of skin, if other products (such as menthol or camphor) are used concurrently, or if an occlusive dressing is used.^5^,^14^,^15^ Scrotal skin has been shown to have up to a 40-fold greater absorption of certain substances compared to other dermal regions.^16^

There are websites and internet-based discussion forums describing the use of various products, including those containing methylsalicylate, to enhance sensation during male masturbation. Emergency physicians, as part of our practice awareness, have to be knowledgeable of uncommon presentations of common diseases, popular culture and practices, and practices within subcultures. This includes the knowledge of the potentially dangerous use of methylsalicylate-containing rubefacients as described in this case report. The case we report may also have implications in sports medicine. Because methylsalicylate-containing rubefacients are used to treat muscle aches and pains, trainers and sports physicians may consider cautioning athletes to avoid scrotal contamination with methylsalicylate-containing rubefacients when treating upper leg and groin muscle injuries.

We present a unique case of salicylate poisoning resulting from the use of a methylsalicylate-containing rubefacient in a teenaged boy to facilitate masturbation. Saliclyate toxicity has not previously been reported from the genital exposure to methylsaliclyate.

## Figures and Tables

**Table:** Over-the-counter products containing methylsalicylate.^17^

Product name	Methylsalicylate %
Ultra strength BENGAY® cream	30
Greaseless BENGAY® pain relieving cream	15
Maximum strength Flexall® plus pain relieving gel	10
ICY HOT® pain relieving cream	30
ICY HOT® pain relieving stick	30
ICY HOT® pain relieving balm	29
Thera-Gesic® maximum strength pain relieving creme	15
